# (*Z*)-2-[2-(4-Methyl­benzyl­idene)hydrazin­yl]pyridine

**DOI:** 10.1107/S1600536810052372

**Published:** 2010-12-18

**Authors:** Haldorai Yuvaraj, S. Sundaramoorthy, D. Velmurugan, Rajesh G. Kalkhambkar

**Affiliations:** a School of Display and Chemical Engineering, Yeungnam University, Gyeongsan, Gyeongbuk 712-749, Republic of Korea; b Centre of Advanced Study in Crystallography and Biophysics, University of Madras, Guindy Campus, Chennai 600 025, India; c Department of Chemistry, Karnatak Universitys Karnatak Science College, Dharwad 580 001, Karnataka, India

## Abstract

Mol­ecules of the title compound, C_13_H_13_N_3_, are essentially planar (r.m.s. deviation for all non-H atoms = 0.054 Å). The dihedral angle between the two aromatic rings is 6.33 (5)°. In the crystal, pairs of centrosymmetrically related mol­ecules are linked through N—H⋯N hydrogen bonds, forming N—H⋯N dimers with graph-set motif *R_2_^2^*(8).

## Related literature

For the biological activity of hydrazone derivatives, see: Savini *et al.* (2002 ▶); Silva *et al.* (2004 ▶). For a related structure, see: Yuvaraj *et al.* (2010 ▶). For hydrogen-bond motifs, see: Bernstein *et al.* (1995 ▶).
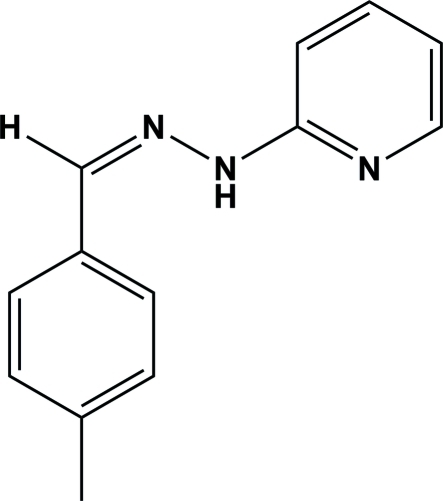


## Experimental

### 

#### Crystal data

C_13_H_13_N_3_
*M* *_r_* = 211.26
Monoclinic, 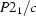

*a* = 5.2385 (8) Å
*b* = 10.7215 (17) Å
*c* = 20.590 (3) Å
β = 92.699 (5)°
*V* = 1155.2 (3) Å^3^
*Z* = 4
Mo *K*α radiation
μ = 0.08 mm^−1^
*T* = 293 K
0.26 × 0.23 × 0.21 mm

#### Data collection

Bruker SMART APEXII area-detector diffractometer
Absorption correction: multi-scan (*SADABS*; Bruker, 2008 ▶) *T*_min_ = 0.981, *T*_max_ = 0.984
10622 measured reflections
2850 independent reflections
1341 reflections with *I* > 2σ(*I*)
*R*_int_ = 0.027

#### Refinement

*R*[*F* ^2^ > 2σ(*F* ^2^)] = 0.045
*wR*(*F* ^2^) = 0.154
*S* = 1.00
2850 reflections
147 parameters
H-atom parameters constrained
Δρ_max_ = 0.14 e Å^−3^
Δρ_min_ = −0.11 e Å^−3^

### 

Data collection: *APEX2* (Bruker, 2008 ▶); cell refinement: *SAINT* (Bruker, 2008 ▶); data reduction: *SAINT*; program(s) used to solve structure: *SHELXS97* (Sheldrick, 2008 ▶); program(s) used to refine structure: *SHELXL97* (Sheldrick, 2008 ▶); molecular graphics: *ORTEP-3* (Farrugia, 1997 ▶); software used to prepare material for publication: *SHELXL97* and *PLATON* (Spek, 2009 ▶).

## Supplementary Material

Crystal structure: contains datablocks global, I. DOI: 10.1107/S1600536810052372/bt5435sup1.cif

Structure factors: contains datablocks I. DOI: 10.1107/S1600536810052372/bt5435Isup2.hkl

Additional supplementary materials:  crystallographic information; 3D view; checkCIF report

## Figures and Tables

**Table 1:** Hydrogen-bond geometry (Å, °)

*D*—H⋯*A*	*D*—H	H⋯*A*	*D*⋯*A*	*D*—H⋯*A*
N2—H2*A*⋯N3^i^	0.86	2.28	3.131 (2)	170

Symmetry code: (i) 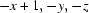.

**Fig. 1.**
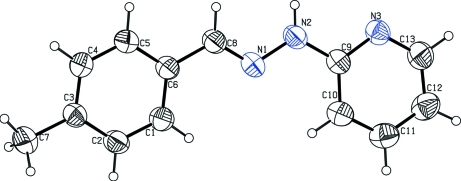
Perspective view of the molecule showing the displacement ellipsoids drawn at 30% probability level.

**Fig. 2.**
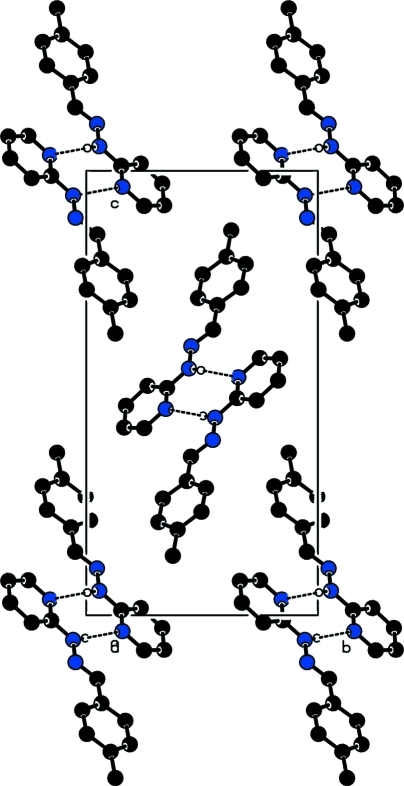
The crystal packing of the molecules viewed down a axis. For clarity, hydrogen atoms which are not involved in hydrogen bonding are omitted

## supplementary crystallographic information

### Comment

The title compound was prepared as part of our continuing interest on the nitrogen based heterocycles (Yuvaraj *et al.*, 2010).

In the title molecule, the C2—C3—C7 and C8—N1—N2 bond angles are 121.65 (2)° and 117.46 (2)°, respectively. The benzene and pyridine  form a dihedral angle of 6.33 (5)°.

In the crystal structure,the molecules at (*x*, *y*, *z*) and (1 - *x*,-*y*,-*z*) are linked by N(2)—H(2 A)···N(3) hydrogen bonds, generating a centrosymmetric dimeric ring motif *R_2_^2^*(8) (Bernstein *et al.*, 1995). The centroid of the *R_2_^2^*(8) motif lies at (1/2,0,0). In addition, there is a weak C—H···N interaction linking the centrosymmetric pair of molecules.

### Experimental

A mixture of 2-hydrazinopyridine and *p*-tolualdehyde were refluxed in ethanol with a catalytic quantity of con. HCl or gl. AcOH. After the reaction was over, the contents were cooled down and filtered to form the product. Diffraction quality crystals were obtained upon recrystallization in ethanol.

### Refinement

H atoms were positioned geometrically (N—H = 0.86 Å, C—H = 0.93–0.98 Å) and allowed to ride on their parent atoms, with 1.5*U*_eq_(C) for methyl H or 1.2 *U*_eq_(C,N) for other H atoms.

### Crystal data

**Table:**

C_13_H_13_N_3_	*F*(000) = 448
*M**_r_* = 211.26	*D*_x_ = 1.215 Mg m^−^^3^
Monoclinic, *P*2_1_/*c*	Mo *K*α radiation, λ = 0.71073 Å
Hall symbol: -P 2ybc	Cell parameters from 715 reflections
*a* = 5.2385 (8) Å	θ = 2.0–28.4°
*b* = 10.7215 (17) Å	µ = 0.08 mm^−^^1^
*c* = 20.590 (3) Å	*T* = 293 K
β = 92.699 (5)°	Block, colourless
*V* = 1155.2 (3) Å^3^	0.26 × 0.23 × 0.21 mm
*Z* = 4	

### Data collection

**Table:**

Bruker SMART APEXII area-detector diffractometer	2850 independent reflections
Radiation source: fine-focus sealed tube	1341 reflections with *I* > 2σ(*I*)
graphite	*R*_int_ = 0.027
ω and φ scans	θ_max_ = 28.4°, θ_min_ = 2.0°
Absorption correction: multi-scan (*SADABS*; Bruker, 2008)	*h* = −6→6
*T*_min_ = 0.981, *T*_max_ = 0.984	*k* = −12→14
10622 measured reflections	*l* = −27→27

### Refinement

**Table:**

Refinement on *F*^2^	Secondary atom site location: difference Fourier map
Least-squares matrix: full	Hydrogen site location: inferred from neighbouring sites
*R*[*F*^2^ > 2σ(*F*^2^)] = 0.045	H-atom parameters constrained
*wR*(*F*^2^) = 0.154	*w* = 1/[σ^2^(*F*_o_^2^) + (0.0649*P*)^2^ + 0.0987*P*] where *P* = (*F*_o_^2^ + 2*F*_c_^2^)/3
*S* = 1.00	(Δ/σ)_max_ < 0.001
2850 reflections	Δρ_max_ = 0.14 e Å^−^^3^
147 parameters	Δρ_min_ = −0.11 e Å^−^^3^
0 restraints	Extinction correction: *SHELXL97* (Sheldrick, 2008), Fc^*^=kFc[1+0.001xFc^2^λ^3^/sin(2θ)]^-1/4^
Primary atom site location: structure-invariant direct methods	Extinction coefficient: 0.011 (3)

### Special details

**Table:**

Geometry. All e.s.d.'s (except the e.s.d. in the dihedral angle between two l.s. planes) are estimated using the full covariance matrix. The cell e.s.d.'s are taken into account individually in the estimation of e.s.d.'s in distances, angles and torsion angles; correlations between e.s.d.'s in cell parameters are only used when they are defined by crystal symmetry. An approximate (isotropic) treatment of cell e.s.d.'s is used for estimating e.s.d.'s involving l.s. planes.
Refinement. Refinement of *F*^2^ against ALL reflections. The weighted *R*-factor *wR* and goodness of fit *S* are based on *F*^2^, conventional *R*-factors *R* are based on *F*, with *F* set to zero for negative *F*^2^. The threshold expression of *F*^2^ > σ(*F*^2^) is used only for calculating *R*-factors(gt) *etc*. and is not relevant to the choice of reflections for refinement. *R*-factors based on *F*^2^ are statistically about twice as large as those based on *F*, and *R*- factors based on ALL data will be even larger.

### Fractional atomic coordinates and isotropic or equivalent isotropic displacement parameters (Å^2^)

**Table:**

	*x*	*y*	*z*	*U*_iso_*/*U*_eq_	
C1	−0.2431 (4)	0.01692 (17)	0.21369 (9)	0.0756 (5)	
H1	−0.2750	0.0866	0.1877	0.091*	
C2	−0.3858 (4)	−0.00253 (17)	0.26709 (9)	0.0797 (5)	
H2	−0.5119	0.0548	0.2766	0.096*	
C3	−0.3465 (3)	−0.10558 (17)	0.30708 (8)	0.0712 (5)	
C4	−0.1622 (4)	−0.18826 (18)	0.29040 (9)	0.0852 (6)	
H4	−0.1341	−0.2594	0.3156	0.102*	
C5	−0.0171 (4)	−0.16896 (18)	0.23728 (9)	0.0874 (6)	
H5	0.1077	−0.2269	0.2276	0.105*	
C6	−0.0525 (3)	−0.06569 (15)	0.19808 (8)	0.0674 (5)	
C7	−0.5002 (4)	−0.1260 (2)	0.36606 (9)	0.0936 (6)	
H7A	−0.4823	−0.2110	0.3801	0.140*	
H7B	−0.6770	−0.1086	0.3553	0.140*	
H7C	−0.4395	−0.0714	0.4003	0.140*	
C8	0.1072 (3)	−0.04777 (17)	0.14289 (8)	0.0731 (5)	
H8	0.2328	−0.1063	0.1347	0.088*	
C9	0.1978 (3)	0.15130 (15)	0.01140 (8)	0.0662 (5)	
C10	−0.0022 (4)	0.23520 (18)	0.01634 (9)	0.0813 (5)	
H10	−0.1154	0.2281	0.0496	0.098*	
C11	−0.0290 (4)	0.32800 (19)	−0.02841 (10)	0.0907 (6)	
H11	−0.1607	0.3857	−0.0258	0.109*	
C12	0.1387 (4)	0.33637 (19)	−0.07751 (11)	0.0951 (6)	
H12	0.1231	0.3990	−0.1087	0.114*	
C13	0.3290 (4)	0.2497 (2)	−0.07891 (10)	0.0895 (6)	
H13	0.4430	0.2554	−0.1121	0.107*	
N1	0.0784 (3)	0.04629 (14)	0.10557 (6)	0.0720 (4)	
N2	0.2388 (3)	0.05650 (13)	0.05528 (6)	0.0749 (4)	
H2A	0.3623	0.0046	0.0513	0.090*	
N3	0.3633 (3)	0.15655 (13)	−0.03568 (7)	0.0742 (4)	

### Atomic displacement parameters (Å^2^)

**Table:**

	*U*^11^	*U*^22^	*U*^33^	*U*^12^	*U*^13^	*U*^23^
C1	0.0777 (12)	0.0642 (10)	0.0860 (12)	0.0007 (9)	0.0162 (9)	0.0076 (9)
C2	0.0744 (12)	0.0739 (12)	0.0927 (12)	0.0054 (10)	0.0244 (10)	0.0042 (10)
C3	0.0665 (11)	0.0751 (11)	0.0727 (10)	−0.0031 (9)	0.0112 (8)	0.0009 (9)
C4	0.0932 (14)	0.0836 (13)	0.0798 (12)	0.0151 (11)	0.0158 (10)	0.0178 (10)
C5	0.0928 (14)	0.0874 (13)	0.0837 (12)	0.0246 (11)	0.0219 (11)	0.0115 (10)
C6	0.0682 (11)	0.0661 (10)	0.0687 (10)	−0.0004 (9)	0.0094 (8)	−0.0022 (8)
C7	0.0926 (14)	0.1023 (15)	0.0878 (13)	0.0005 (12)	0.0244 (11)	0.0100 (11)
C8	0.0754 (12)	0.0720 (11)	0.0729 (10)	0.0023 (9)	0.0137 (9)	−0.0022 (9)
C9	0.0638 (11)	0.0657 (11)	0.0693 (10)	−0.0110 (9)	0.0067 (8)	−0.0058 (8)
C10	0.0767 (12)	0.0795 (12)	0.0884 (12)	0.0016 (11)	0.0117 (10)	−0.0051 (11)
C11	0.0797 (14)	0.0773 (13)	0.1152 (16)	0.0026 (10)	0.0057 (12)	0.0009 (12)
C12	0.0857 (15)	0.0804 (13)	0.1190 (16)	−0.0064 (12)	0.0035 (13)	0.0221 (12)
C13	0.0778 (13)	0.0941 (14)	0.0976 (14)	−0.0129 (12)	0.0151 (10)	0.0157 (12)
N1	0.0709 (9)	0.0764 (10)	0.0699 (8)	−0.0061 (7)	0.0162 (7)	−0.0064 (8)
N2	0.0737 (10)	0.0783 (10)	0.0741 (9)	0.0027 (8)	0.0191 (7)	0.0002 (8)
N3	0.0671 (9)	0.0762 (10)	0.0801 (9)	−0.0092 (7)	0.0115 (8)	0.0036 (8)

### Geometric parameters (Å, °)

**Table:**

C1—C2	1.374 (2)	C8—N1	1.273 (2)
C1—C6	1.383 (2)	C8—H8	0.9300
C1—H1	0.9300	C9—N3	1.332 (2)
C2—C3	1.387 (2)	C9—N2	1.370 (2)
C2—H2	0.9300	C9—C10	1.388 (2)
C3—C4	1.366 (2)	C10—C11	1.359 (2)
C3—C7	1.504 (2)	C10—H10	0.9300
C4—C5	1.377 (3)	C11—C12	1.373 (3)
C4—H4	0.9300	C11—H11	0.9300
C5—C6	1.378 (2)	C12—C13	1.364 (3)
C5—H5	0.9300	C12—H12	0.9300
C6—C8	1.455 (2)	C13—N3	1.344 (2)
C7—H7A	0.9600	C13—H13	0.9300
C7—H7B	0.9600	N1—N2	1.3680 (17)
C7—H7C	0.9600	N2—H2A	0.8600
			
C2—C1—C6	120.95 (17)	N1—C8—C6	121.34 (17)
C2—C1—H1	119.5	N1—C8—H8	119.3
C6—C1—H1	119.5	C6—C8—H8	119.3
C1—C2—C3	121.63 (17)	N3—C9—N2	115.09 (16)
C1—C2—H2	119.2	N3—C9—C10	123.05 (17)
C3—C2—H2	119.2	N2—C9—C10	121.86 (17)
C4—C3—C2	117.02 (16)	C11—C10—C9	118.63 (18)
C4—C3—C7	121.33 (17)	C11—C10—H10	120.7
C2—C3—C7	121.65 (17)	C9—C10—H10	120.7
C3—C4—C5	121.69 (18)	C10—C11—C12	119.8 (2)
C3—C4—H4	119.2	C10—C11—H11	120.1
C5—C4—H4	119.2	C12—C11—H11	120.1
C4—C5—C6	121.46 (18)	C13—C12—C11	117.68 (19)
C4—C5—H5	119.3	C13—C12—H12	121.2
C6—C5—H5	119.3	C11—C12—H12	121.2
C5—C6—C1	117.22 (16)	N3—C13—C12	124.55 (19)
C5—C6—C8	119.79 (16)	N3—C13—H13	117.7
C1—C6—C8	122.99 (16)	C12—C13—H13	117.7
C3—C7—H7A	109.5	C8—N1—N2	117.46 (15)
C3—C7—H7B	109.5	N1—N2—C9	118.41 (15)
H7A—C7—H7B	109.5	N1—N2—H2A	120.8
C3—C7—H7C	109.5	C9—N2—H2A	120.8
H7A—C7—H7C	109.5	C9—N3—C13	116.24 (17)
H7B—C7—H7C	109.5		
			
C6—C1—C2—C3	−0.5 (3)	N3—C9—C10—C11	−0.8 (3)
C1—C2—C3—C4	−1.1 (3)	N2—C9—C10—C11	179.07 (15)
C1—C2—C3—C7	179.13 (16)	C9—C10—C11—C12	0.6 (3)
C2—C3—C4—C5	1.6 (3)	C10—C11—C12—C13	−0.3 (3)
C7—C3—C4—C5	−178.62 (18)	C11—C12—C13—N3	0.2 (3)
C3—C4—C5—C6	−0.6 (3)	C6—C8—N1—N2	179.29 (13)
C4—C5—C6—C1	−1.0 (3)	C8—N1—N2—C9	174.71 (14)
C4—C5—C6—C8	179.16 (17)	N3—C9—N2—N1	179.08 (13)
C2—C1—C6—C5	1.5 (3)	C10—C9—N2—N1	−0.8 (2)
C2—C1—C6—C8	−178.67 (16)	N2—C9—N3—C13	−179.22 (14)
C5—C6—C8—N1	179.65 (16)	C10—C9—N3—C13	0.7 (2)
C1—C6—C8—N1	−0.2 (3)	C12—C13—N3—C9	−0.3 (3)

### Hydrogen-bond geometry (Å, °)

**Table:**

*D*—H···*A*	*D*—H	H···*A*	*D*···*A*	*D*—H···*A*
N2—H2A···N3^i^	0.86	2.28	3.131 (2)	170

Symmetry codes: (i) −*x*+1, −*y*, −*z*.
